# MRI Characteristics of Pediatric and Young-Adult Renal Cell Carcinoma: A Single-Center Retrospective Study and Literature Review

**DOI:** 10.3390/cancers15051401

**Published:** 2023-02-22

**Authors:** Justine N. van der Beek, Ronald R. de Krijger, Rutger A. J. Nievelstein, Axel Bex, Aart J. Klijn, Marry M. van den Heuvel-Eibrink, Annemieke S. Littooij

**Affiliations:** 1 Department of Radiology and Nuclear Medicine, University Medical Center Utrecht/Wilhelmina Children’s Hospital, Utrecht University, 3584 CX Utrecht, The Netherlands; 2 Princess Máxima Center for Pediatric Oncology, 3584 CS Utrecht, The Netherlands; 3 Department of Pathology, University Medical Center Utrecht, 3584 CX Utrecht, The Netherlands; 4 Department of Urology, The Netherlands Cancer Institute, Antoni van Leeuwenhoek Hospital, 1066 CX Amsterdam, The Netherlands; 5 Division of Surgical and Interventional Science, The Royal Free London NHS Foundation Trust and UCL, London NW3 2QG, UK; 6 Department of Pediatric Urology, University Medical Center Utrecht/Wilhelmina Children’s Hospital, 3584 CX Utrecht, The Netherlands

**Keywords:** renal cell carcinoma, magnetic resonance imaging, pediatrics, radiology, pathology

## Abstract

**Simple Summary:**

Whereas renal cell carcinoma (RCC) is the most common renal tumor in adults, pediatric RCC is a rare malignancy. The previous literature focusing on cross-sectional imaging of RCC concerns mainly computed tomography in adults, whereas in children, a different distribution of subtypes is seen, as well as a preference for magnetic resonance imaging (MRI). Therefore, the aim of this study was to identify MRI characteristics of pediatric and young-adult RCC through a case series and literature review focusing on translocation-type RCC (MiT-RCC) and the pediatric and young-adult population. In our review as well as in our case series T2-weighted hypo-intensity seems to be a potential discriminative characteristic. Moreover, an irregular growth pattern and limited diffusion restriction were often described. Nevertheless, we conclude the discrimination of RCC subtypes, and especially the differentiation of RCC from other pediatric renal tumors, remains difficult.

**Abstract:**

Pediatric renal cell carcinoma (RCC) is a rare malignancy. Magnetic resonance imaging (MRI) is the preferred imaging modality for assessment of these tumors. The previous literature has suggested that cross-sectional-imaging findings differ between RCC and other pediatric renal tumors and between RCC subtypes. However, studies focusing on MRI characteristics are limited. Therefore, this study aims to identify MRI characteristics of pediatric and young-adult RCC, through a single-center case series and literature review. Six identified diagnostic MRI scans were retrospectively assessed, and an extensive literature review was conducted. The included patients had a median age of 12 years (63–193 months). Among other subtypes, 2/6 (33%) were translocation-type RCC (MiT-RCC) and 2/6 (33%) were clear-cell RCC. Median tumor volume was 393 cm^3^ (29–2191 cm^3^). Five tumors had a hypo-intense appearance on T2-weighted imaging, whereas 4/6 were iso-intense on T1-weighted imaging. Four/six tumors showed well-defined margins. The median apparent diffusion coefficient (ADC) values ranged from 0.70 to 1.20 × 10^−3^ mm^2^/s. In thirteen identified articles focusing on MRI characteristics of MiT-RCC, the majority of the patients also showed T2-weighted hypo-intensity. T1-weighted hyper-intensity, irregular growth pattern and limited diffusion–restriction were also often described. Discrimination of RCC subtypes and differentiation from other pediatric renal tumors based on MRI remains difficult. Nevertheless, T2-weighted hypo-intensity of the tumor seems a potential distinctive characteristic.

## 1. Introduction

Pediatric renal cell carcinoma (RCC) is a rare renal malignancy [1,2]. Although Wilms tumors (WTs) show the highest prevalence in young children, the incidence of RCC increases in the second decade of life [1,3,4]. Whereas in the Renal Tumor Study Group of the International Society of Pediatric Oncology (SIOP-RTSG), pre-operative chemotherapy is the standard of care for WTs, upfront surgery is recommended for localized RCC [5]. Invasive procedures to determine histology before the start of therapy in young children are discouraged [6,7]. Age and size of the tumor are important factors in the consideration of the diagnosis of pediatric renal tumors as well as in the consideration of performing a biopsy, indicating age >7 years as a criterion to consider tumor biopsy [6]. Thus far, no specific imaging characteristics discriminating RCC from WTs and other non-WTs have been identified [8,9,10].

Magnetic resonance imaging (MRI) is currently the preferred modality for the assessment of pediatric renal tumors within the SIOP-RTSG given its lack of ionizing radiation and excellent soft-tissue contrast. Furthermore, MRI is subject to continuous technical developments, such as the possibility of calculating the apparent diffusion coefficient (ADC) value using diffusion-weighted imaging (DWI) [6,11,12]. MRI could, therefore, play a potential role in the non-invasive discrimination of pediatric renal tumors [13,14,15,16,17].

Contrary to the rarity of RCC in children, this tumor type is the most common renal tumor in adolescents and adults [18,19,20,21]. Nevertheless, childhood RCC shows distinct histological characteristics, possibly related to the different distribution of RCC subtypes. Whereas translocation-type RCC (MiT-RCC), which has been officially recognized since 2004 by the World Health Organization, is the most frequent subtype in children, clear-cell RCC (ccRCC) is the predominant histological subtype in adults [2,5,22,23,24,25]. MiT-RCC is diagnosed based on translocations including transcription factor E3 (TFE3) and EB (TFEB), which are members of the family of microphthalmia transcription factors (MiT) [26,27]. Interestingly, the previous literature has suggested that cross-sectional imaging findings differ between RCC subtypes [13,28,29,30,31,32,33,34,35].

Until today, studies focusing on the MRI characteristics of pediatric RCC are limited in number, although identification of potential specific MRI characteristics of WTs and non-WTs is important for future validation studies [9,25,35]. Therefore, this study aims to retrospectively identify MRI characteristics of pediatric RCC patients at diagnosis through a case series in our center, including a literature review focusing on this topic.

## 2. Materials and Methods

### 2.1. Patients

Institutional Review Board approval was obtained. For this retrospective study, obtaining further formal consent was waived. All diagnostic MRI scans included were clinically indicated and were performed as the standard of care. Between 2014 and 2019, we identified 6 children with RCC that underwent an MRI scan at diagnosis.

The standard of care for localized pediatric RCC is upfront total nephrectomy [22,36]. Only in case of doubt of a WT diagnosis, based on predefined clinical and imaging characteristics, a core needle biopsy was performed. If there was no suspicion of a non-WT, the patients were pre-operatively treated with 4 weeks of vincristine/actinomycin-D (stage I-III) or 6 weeks of vincristine/actinomycin-D/doxorubicin (stage IV/V), according to the SIOP-RTSG protocol.

### 2.2. Magnetic Resonance Imaging Acquisition

Abdominal MRI for pediatric renal tumors in this study was performed using a 1.5T MRI system (Achieva, Philips Healthcare, Eindhoven, The Netherlands and Ingenia, Philips Healthcare, Eindhoven, The Netherlands). Two patients were scanned in external hospitals at diagnosis before referral to our center (Signa HDxt; GE Healthcare, Boston, USA and Magnetom Avanto; Siemens, Erlangen, Germany). Scan protocols slightly varied but at least consisted of coronal and axial T2-weighted imaging, axial T1-weighted turbo spin-echo and axial DWI with automatically generated ADC maps. Five patients underwent pre- and post-contrast T1-weighted imaging, whereas for one patient, contrast-enhanced MRI was not available (Table 1).

Children were awake, sedated or under general anesthesia depending on their ability to cooperate, according to the standard-of-care procedures. Gadobutrol (Gadovist; Bayer B.V., Leverkusen, Germany) was administered intravenously at a dose of 0.1 mL/kg body weight. Hyoscine butylbromide (Buscopan; Sanofi, Paris, France) was administered intravenously at a dose of 0.4 mg/kg body weight to reduce peristaltic artifacts, with a maximum of 10 mg in children ≥6 years and a maximum of 5 mg in children <6 years. All children were screened for contraindications for MRI and those concerning intravenous agents. For the two patients scanned at local hospitals, specifications of gadobutrol and hyoscine butylbromide were not available.

### 2.3. Image Analysis

The anonymized MRI datasets were transferred to DICOM software Osirix v. 5.5.2 (Pixmero, SARL, Bernex, Switzerland). Two pediatric radiologists (ASL with 13 years of experience and RAJN with 26 years of experience in body MRI, respectively), who were blinded to the histopathological subtype and clinical characteristics but were aware of the pediatric RCC diagnosis, reviewed the diagnostic MRI scans. All diagnostic scans were assessed using a case report form based on previous studies identifying potential specific imaging characteristics of different pediatric renal tumors [9]. The pediatric RCC cases were analyzed focusing on tumor presentation, growth pattern, characteristics of solid components and enhancement pattern, if available. Tumor volume was calculated based on the three dimensions of the tumor times 0.523. Moreover, up to four round-shaped ROIs containing solid areas of the tumor, mainly based on enhancement, were drawn in order to measure the ADC value of the most representative parts of the tumor. To limit inter-observer variability, an instruction form accompanying the case report form was provided.

### 2.4. Histopathological Review

Our national coordinating SIOP-RTSG histopathologist (RRK with 23 years of experience with pediatric renal tumor histopathology) reviewed the available macroscopy and microscopy from the surgically resected tumors and biopsies of all patients following the most recent WHO classification system [27,37].

Following protocol, the dorsal and ventral side and hilar region of the resected specimen were marked with varying color dyes following the instructions of the involved surgeons. The specimens were sliced free-handed in successive 10 to 20 mm transverse macroscopic slices in a cranial to caudal sequence or through longitudinal incision to bivalve the specimen.

### 2.5. Statistical Analyses

Due to the small number of patients, inter-observer agreement between the two pediatric radiologists was difficult to assess because Cohen’s kappa is affected by the prevalence of the finding under observation. Only six patients were included in this study, potentially resulting in low values or even an impossible calculation of kappa when focusing on separate characteristics [38,39]. Therefore, the inter-observer agreement was assessed using percentages of observed agreement, including the intra-class correlation coefficient (ICC) for the median ADC values including the regions of interest and for median tumor volumes. ICC values were interpreted as satisfactory >0.75 [40].

### 2.6. Literature Review

A literature review was performed following PRISMA guidelines to reflect on the case series and elaborate on the current knowledge about the MRI appearance of RCC by focusing on the predominant histological subtypes in the pediatric and young-adult population. For this purpose, PubMed, Embase/Medline and Cochrane libraries were searched in November 2021, using the main search terms ‘renal cell carcinoma’ and ‘magnetic resonance imaging’ (Table S1). The study has not been registered. Cross-referencing and a citation check of the included papers were executed using Scopus.

**Figure 1:**
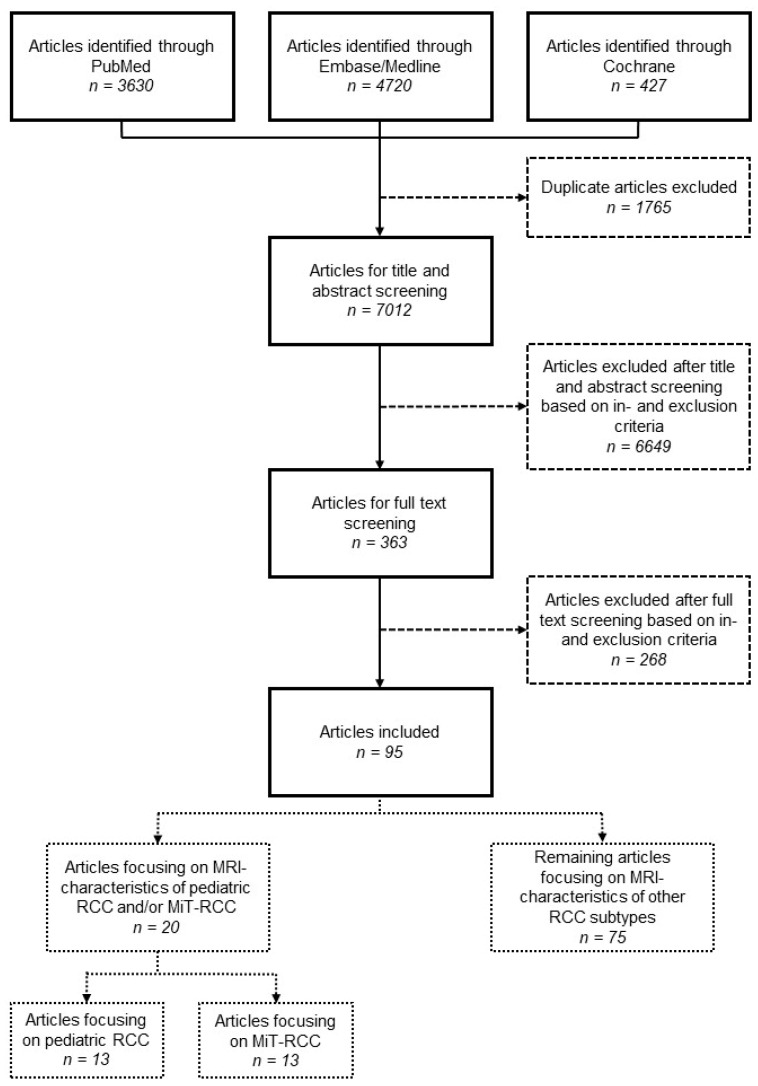
Flow chart of the literature review.

Articles were included when they (1) included MRI characteristics of patients with proven RCC; (2) were prospective or retrospective cohort studies, randomized controlled trials or case reports; (3) were written in the English language; and (4) were available in the full-text form. Subsequently, articles focusing on children (<19 years), potentially also including adolescents or young adults (≤35 years) and articles focusing on MiT-RCC were separated to serve as the focus of this literature review. Given the rarity of studies focusing on the MRI characteristics of MiT-RCC, articles focusing on adults were also included for this purpose. With this approach, we guaranteed identification of all relevant articles while subdividing their relevance for our study based on their full-text content. After removal of duplicates, 7012 articles were screened based on title and abstract, leaving 363 articles for full-text screening, resulting in the inclusion of 95 articles. Of these, 13 articles focused on pediatric, adolescent and young-adult RCC, and 13 articles focused on MiT-RCC, with an overlap of 6 articles (Figure 1). In November 2022, the search was updated, with no additional results for articles focusing on children and/or MiT-RCC.

## 3. Results

### 3.1. Case Presentation

#### 3.1.1. Patient Characteristics

The six identified patients in our center had a median age of 12 years (range 63–193 months) (Table 2). Four patients were female, and half of the patients presented with a right-sided tumor. Two/six patients received pre-operative chemotherapy following suspicion of a WT, whereas 4/6 underwent upfront surgery. In one case, RCC was pre-operatively confirmed through tumor biopsy. Three patients had stage 1 disease, whereas the other patients had stage 2 (1/6) and stage 3 (2/6) disease (Table 2).

#### 3.1.2. Histopathology

The average post-operative specimen weight was 898.6 g (range 210–2100 g), whereas the maximum post-operative tumor diameter ranged from 2.4 to 12.9 cm (median 6.8 cm). The post-operative weight of the specimen was missing for one patient, of which the largest tumor diameter was 9.5 cm (Table 2).

**Figure 2:**
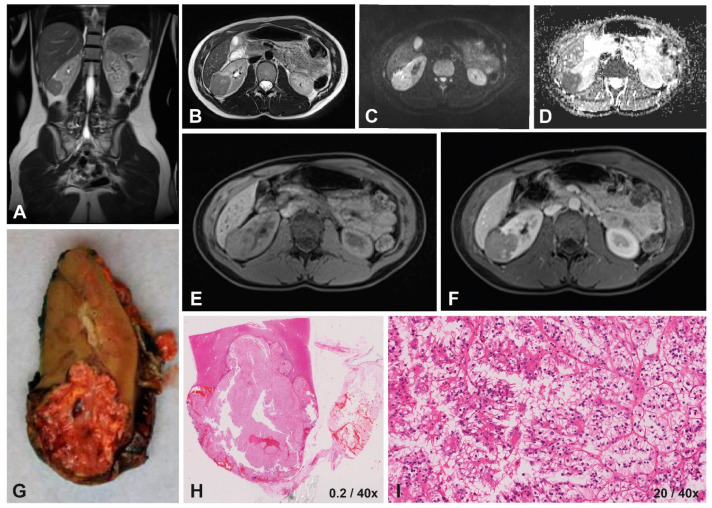
Imaging and histopathology of a 14-year-old female patient with a right-sided translocation-type RCC (MiT-RCC). On T2-weighted imaging (**A**,**B**) the tumor appears hypo-intense with ill-defined margins, compared to the iso-intense homogeneous appearance on T1-weighted imaging (**E**) with relatively strong homogeneous enhancement on T1-weighted contrast-enhanced imaging (**F**). DWI showed restricted diffusion on the b500 scan (**C**), with a relatively high median ADC value of 0.98x10^−3^ mm^2^/s calculated based on the b0/b500s map (**D**). The macroscopic (**G**) and microscopic histopathology (**H**) showed an infiltrating tumor, detail showing tumor cells with hyperchromatic nuclei and papillary growth pattern (**I**).

**Figure 3:**
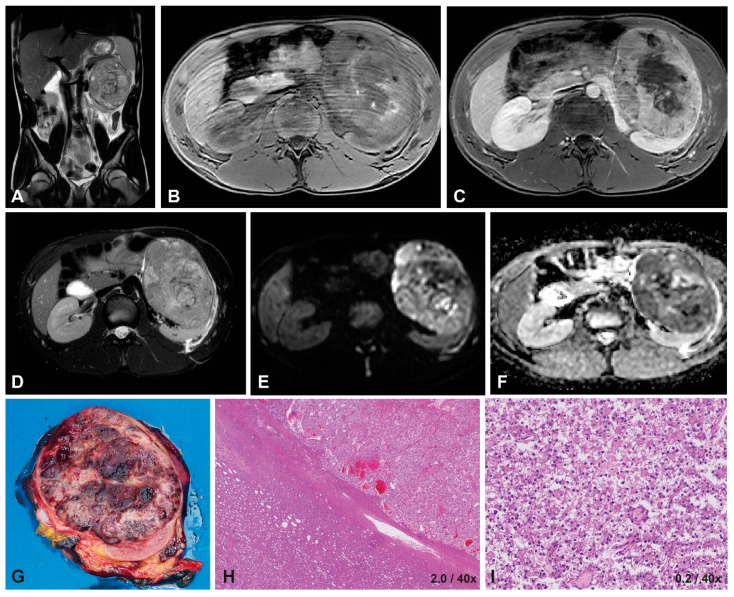
Imaging and histopathology of a 16-year-old male patient with a left-sided translocation-type RCC (MiT-RCC). On T2-weighted imaging (**A**,**D**) the tumor appears hypo-intense and heterogeneous with well-defined margins, similar to a hypo-intense appearance on T1-weighted imaging (**B**) with mild, heterogeneous enhancement on T1-weighted contrast-enhanced imaging (**C**). DWI showed restricted diffusion on the b1000 scan (**E**), with a median ADC value of 0.80 ×10^−3^ mm^2^/s on the b0/b1000 map (**F**). The macroscopic histopathology (**G**) shows a large, round tumor, with little remaining normal renal tissue. The microscopic HE image (**H**) shows a capsule around the tumor, with a predominantly epithelial growth pattern in nests, often with cells with clear cytoplasm and mildly atypical nuclei (**I**).

Five patients were tested for MiT-RCC, resulting in 2/5 MiT-RCC cases (Table 2, Figure 2 and Figure 3). In 4/5 cases, FISH was used, whereas in the two most recent cases, also RNA sequencing was performed, resulting in a rearrangement of TFE3 and SFPQ in the sixth patient. Two patients were diagnosed with ccRCC, and in one patient, the subtype could not be specified. The first patient, who was not tested for MiT-RCC, showed an FH mutation in the context of a hereditary leiomyomatosis and RCC cancer syndrome (Table 2) [41]. For the 5-year-old patient diagnosed with ccRCC, the FISH for MiT-RCC was not conclusive, and RNA sequencing for further analysis of TFEB was not available.

#### 3.1.3. Imaging Characteristics at Diagnosis

The median observed agreement between the two observers was 83% (range 33.3%–100%). The few imaging characteristics with low observed agreement were discussed between the two radiologists, and mismatching concepts were resolved (Table S2). Furthermore, the inter-reader agreement for median tumor volume was excellent, with an ICC of 0.991 (95% 0.941–0.999). Therefore, the imaging characteristics found by the first reader (ASL) were reported (Table 2).

Tumor volume ranged from 29 to 2191 cm^3^, with varying locations. The shape of the tumors was predominantly lobulated (4/6), and margins were well-defined in a majority of the patients (4/6). Capsule rupture was seen in only 2/6 cases, which was defined as an interruption of the hypo-intense capsule of the tumor. None of the cases presented with a tumor thrombus. Concerning hemorrhage and necrosis, these components were present in 3/6 and 1/6 cases, respectively. Cysts were present in 2/6 cases, whereas fatty tissue and subcapsular fluid were not observed (Table 2).

The tumors presented mainly homogeneously (4/6), with a predominant hypo-intense appearance on T2-weighted imaging and iso-intense appearance on T1-weighted imaging. Almost all cases showed a homogeneous enhancement pattern, varying from mild to strong enhancement (Table 2). There was no obvious consistency concerning MRI characteristics within patients based on histological subtype (Table 2, Figure 2, Figure 3).

#### 3.1.4. Diffusion-Weighted Imaging

Inter-reader agreement was excellent for median ADC values with an ICC of 0.942 (95% CI 0.639–0.992) (Table S3). Therefore, only the median surfaces of ROIs and median ADC values measured by the first reader (ASL) were reported (Table 2). The median ADC values ranged from 0.70 to 1.20 × 10^−3^ mm^2^/s. The MiT-RCC cases and the case diagnosed as ccRCC but with inconclusive TFE results showed the lowest ADC values, ranging from 0.70 to 0.98 × 10^−3^ mm^2^/s (Table 2, Figure 2 and Figure 3).

### 3.2. Literature Review

#### 3.2.1. Pediatric and Young-Adult RCC

We identified thirteen studies focusing on MRI findings of pediatric RCC, with a total of 25 patients (Figure 1, Table 3) [19,24,42,43,44,45,46,47,48,49,50,51,52]. Ages ranged from 4 to 33 years, with four studies also including young adults ≤35 years [19,24,48,51]. Six studies focused on MiT-RCC, whereas other histological subtypes represented ccRCC, papillary type RCC (pRCC), chromophobe RCC (chrRCC), renal medullary carcinoma (RMC) and other rare RCC types.

The location of all reported pediatric RCC tumors in the identified articles varied from central to peripheral (Table 3). On T1-weighted imaging and T2-weighted imaging, tumors appeared predominantly heterogeneously, whereas no clear predominant intensity was seen for one of these sequences. Accordingly, enhancement pattern on contrast-enhanced MRI was reported mostly as heterogeneous. Cysts, when specified, were found in only three cases, whereas the presence of necrosis and/or hemorrhage was often not specified [24,43,52].

Regional lymph node involvement and/or metastases to lymph nodes were reported in five studies (Table 3) [19,24,42,43,51]. In a study of seven patients, Wang et al. reported positive regional lymph node status in four patients and positive cervical lymph node status in one patient [19]. Blitman et al. reported two patients with vascular tumor involvement of the renal vein and three patients with encasement of the vascular pedicle out of a total of six patients, all with infiltrative tumors with ill-defined margins (Table 3) [51]. Only one study specified findings of DWI, reporting the iso-intense appearance of the tumor on the b500 DWI sequence compared to the renal parenchyma [50].

Concerning MRI characteristics of RCC subtypes other than MiT-RCC in children, Zou et al. reported a case of a 17-year-old male with von Hippel–Lindau disease with bilateral renal cysts and ccRCC (Table 3) [46]. This patient showed T2-weighted hyper-intensity and T1-weighted hypo-intensity, whereas enhancement was limited on contrast-enhanced imaging. Koetter et al. described a 16-year-old female at 31 weeks’ gestation presenting with a large, heterogeneous cystic–solid mass, which was histologically diagnosed as pRCC [43]. Another reported pRCC that presented as a complex cyst containing bloody elements, whereas a pediatric chrRCC showed a well-defined T1-weighted hypo-intense and T2-weighted hyper-intense tumor with necrosis (Table 3) [45,52].

Finally, RMC has been described as a very rare and malignant renal tumor, especially in children and young adults, and is often seen in RCC patients with sickle-cell traits [28,51,53]. Noreña-Rengifo et al. described a 12-year-old male with an intermediate enhancing mass on T1-weighted imaging with evident retroperitoneal lymphadenopathies, similar to the reported regional adenopathy identified on MRI in a retrospective study by Blitman et al. (Table 3) [42,51].

#### 3.2.2. MiT-RCC

Thirteen studies focusing on MRI characteristics of MiT-RCC were identified, including the six identified studies focusing on pediatric patients with MiT-RCC (Figure 1, Table 3 and Table 4) [13,19,24,44,47,48,50,54,55,56,57,58,59]. There was a total of 46 patients, who were aged 4–76 years old, with MiT-RCC included in the identified articles. Whereas the tumor location was again highly variable among patients, overall, there was a majority showing hyper-intensity on T1-weighted imaging and hypo-intensity on T2-weighted imaging, with a heterogeneous enhancement pattern. Wang et al. reported 8/9 patients with necrosis, and 7/9 patients with hemorrhage, whereas in other studies, these characteristics were often not specified [19]. The tumor composition and growth pattern of MiT-RCC was very heterogeneous, although a substantial part of the cases seems to present with an infiltrative and/or irregular growth pattern. Fifteen patients presented with lymph node involvement; however, four studies lacked information concerning this characteristic. Reported metastatic sites were liver and/or lungs in a total of three patients [55,57].

DWI characteristics were reported in 5 studies for a total of 23 patients [13,50,54,55,57]. Overall, diffusion restriction seemed limited in these cases, with, for instance, Tohi et al. reporting no restriction and Chen et al. reporting a relatively high signal on the ADC map [54,57]. Razek et al. showed a mean ADC value of 1.50 ± 0.97 for four patients [13].

In our case series, the 14-year-old female patient in particular showed a typical presentation of MiT-RCC based on these findings in the previous literature. The tumor showed an ill-defined tumor with capsule invasion and an infiltrative growth pattern, appearing hypo-intense on T2-weighted imaging with a relatively high median ADC value (Table 2 and Table 4, Figure 2). The presentation of the 16-year-old male patient with MiT-RCC seemed less typical (Table 2 and Table 4, Figure 3).

#### 3.2.3. Other Subtypes

The RCC subtypes most frequently occurring in children and adolescents besides MiT-RCC are ccRCC, pRCC and chrRCC (Table 3) [3,18]. Knowledge of MRI characteristics of these subtypes is based mainly on adult studies.

A retrospective study of Wang et al. focused on the MRI characteristics of 57 adult RCC patients, in which ccRCC and pRCC showed hemorrhage in 20–25% of the cases compared to no evidence of hemorrhage for chrRCC [60]. Moreover, a very high percentage of cystic necrosis was seen in ccRCC and pRCC, resulting in a significant difference of this characteristic compared to chrRCC, for which no cases were seen. Compared to ccRCC, other RCC subtypes often show a less aggressive growth pattern on MRI, which is illustrated by a higher numbers of cases with well-defined margins, less peripheral invasion and less extension of the tumor [60,61].

Oliva et al. described the MRI-features of 21 pRCCs and 28 ccRCCs, concluding that pRCC typically presents with T2 hypo-intensity, whereas ccRCC typically shows T2 hyper-intensity [62]. This finding, as well as the occurrence of increased enhancement in ccRCC compared to pRCC and chrRCC, has often been reported in the previous literature [35,63,64,65]. Furthermore, ccRCC seems to show significantly higher ADC values than pRCC and chrRCC [64,66,67].

## 4. Discussion

There seems to be a lack of specific imaging characteristics for discrimination of pediatric RCC and its subtypes based on MRI characteristics alone [6,9,10]. Nevertheless, imaging plays an increasingly important role in the diagnosis and follow-up of pediatric renal tumors and in the discrimination of different renal tumor types [28,68,69].

The heterogeneous diagnostic appearance of our patients was in line with findings in the identified literature and with previous studies stating that RCC is often indistinguishable from WTs based on MRI characteristics alone [70,71,72,73]. Part of the included patients showed cysts, necrosis and hemorrhage; however, none of these characteristics were explicitly found in all patients [74]. Calcifications have often been reported as common findings in pediatric RCC; however, MRI does not allow for a trustworthy assessment of calcifications and was, therefore, not included as an imaging characteristic in our case report form [28,69,75]. Despite the recommendation of the SIOP-RTSG to use MRI for cross-sectional imaging of renal tumors, various countries still perform abdominal CT scans in these patients. One of the largest studies focusing on CT characteristics of pediatric RCC to date also reported a widely variable radiological appearance, often with the presence of calcifications [49]. Nevertheless, calcifications can also be seen in WTs, making discrimination based on this imaging characteristic difficult given the rarity of pediatric RCC and other non-WTs [76,77]. Finally, the findings in our case series were in concordance with the frequently reported localized presentation and small size of pediatric RCC [6,75].

Whereas MiT-RCC is the most frequent histological subtype in children, we reported only two out of six patients with a proven TFE translocation. The MRI characteristics of these two patients were quite different from one another. MiT-RCC, similar to ccRCC, is often described as a relatively aggressive tumor in terms of growth pattern and tumor extension as well as prognosis [28,35,60,78,79,80,81]. Nevertheless, only one MiT-RCC case showed an infiltrative growth pattern with capsule rupture, whereas the second MiT-RCC case and both ccRCC cases had well-defined margins with the presence of a pseudocapsule, without any signs of aggressive growth. In general, capsule rupture remains difficult to assess. Concerning the discrimination between histological RCC subtypes, the predominantly reported T2-weighted hypo-intensity in MiT-RCC is also often described for pRCC and chrRCC, whereas ccRCC classically demonstrates high intrinsic T2-weighted signal intensity [31,33,35,82,83]. Nonetheless, knowledge of specific MRI characteristics of MiT-RCC remains limited, given the rarity of MiT-RCC in adult patients and its relatively recent recognition as an official subtype by the WHO [27].

Whereas in adult RCC, the main focus is often the discrimination of histological subtypes, in pediatric RCC, discrimination from the much more frequently occurring WTs in the early diagnostic stages is of great importance [6,7,9]. WTs have a very heterogeneous presentation at diagnosis and are, most often, large intra-renal tumors with a pseudocapsule [74,84,85]. Whereas an irregular growth pattern and absence of a capsule are often described as common for RCC in the previous literature, we observed a majority of well-defined margins and the presence of a pseudocapsule in our case series. Nonetheless, an enhancing capsule has also been reported as a characteristic of MiT-RCC [25,28,57]. MRI characteristics reported to be typical for RCC will still not be discriminative given the heterogeneous appearance of WTs. Nevertheless, WTs often appear hyper-intense on T2-weighted imaging, which is opposite to the T2-weighted hypo-intensity in a majority of our cases with RCC, as substantiated by the findings in the previous literature [28,69]. Finally, RCC is often reported to be smaller than WTs [7,10,57]. Following SIOP-RTSG protocols, based on the suspicion of a non-WT, a biopsy is recommended for children ≥10 years of age and for children between 7 and 10 years old with a tumor volume <200 mL [10]. In our case series, tumor volume was relatively low, except for the expected large FH-RCC case (case nr. 1). In the previous literature, tumor volume ranged widely; however, often only the largest diameter was reported [7,57,74,77,86].

Overall, there seems to remain a lack of pathognomonic MRI characteristics for the discrimination of pediatric RCC from other renal malignancies in children, as well as for the differentiation of histological subtypes [6,9,10]. Nevertheless, DWI has shown an increasing potential reliability for the non-invasive discrimination of renal lesions [15,16,87,88]. Whereas only one included pediatric study focused on the diffusion restriction of pediatric RCCs, our literature review confirmed results from previous overviews stating adult clear-cell RCC has shown significantly higher ADC values compared to non-clear-cell RCC [17,32,50,87,89]. In contrast, our case series showed the three lowest median ADC values in the ccRCC and MiT-RCC cases, whereas also relatively high ADC values were reported. In WTs, relatively low ADC values can be observed, varying among histological WT subtypes [12,16]. In children, discrimination of common histological RCC subtypes, as well as discrimination from WTs based on DWI, therefore, remains difficult. Nonetheless, the female patient with MiT-RCC in our case series appeared to have a typical presentation in the light of previous reports, showing potential discriminative MRI characteristics for TFE-positive tumors. Future studies may focus on validating adult findings in the pediatric population and explore the relationship between ADC values and common pediatric RCC subtypes combined with other typical MRI characteristics.

Over the past decades, differences between adult and pediatric RCC have increasingly been appreciated. Concerning imaging studies, the direct comparison of the pediatric and adult population has become even more complicated by the preference of CT in the adult population, whereas MRI has developed as the preferred imaging modality within the SIOP-RTSG [6,8]. Nevertheless, MRI also plays an increasingly important role in the adult population, mainly due to its ability to perform quantitative measurements [32,90]. Therefore, when searching the literature databases for MRI characteristics of pediatric RCC and MiT-RCC, the literature about the adolescent and adult population cannot be ignored. Not only because knowledge of MR imaging of these cases is scarce, but also because they are often embedded in studies focusing on adolescents and/or adults as well. Concerning cut-off values for age classification, we focused on the predefined range of 18–35 years for the ‘adolescents and young adults’ often used in Europe. However, this classification varies around the world [91,92].

Our study has a few limitations, mainly based on its retrospective nature and small study population. The limited number of patients did not allow any statistical analysis or strong conclusions. Furthermore, scan parameters were inconsistent due to not as yet centralized care. Nevertheless, these cases served mainly as an illustration accompanying the literature review in this developing field of research. In this way, this descriptive study contributes to the increasing knowledge of pediatric RCC and its diagnostic presentation on MRI. Concerning the reported imaging characteristics by two independent observers, there was excellent inter-observer agreement [39,40]. The small number of patients in this study does not allow for strong conclusions concerning validity of the use of the CRF in other populations.

## 5. Conclusions

For a few years, MRI has been the preferred imaging modality for imaging pediatric renal tumors within the SIOP-RTSG protocol. This case series represents one of the largest retrospective reports so far, including an extensive review focusing on MRI characteristics of RCC in the pediatric and young-adult population. The reported cases showed a varying presentation of different pediatric RCC subtypes on MRI, in line with the published literature. Nevertheless, based on this study, T2-weighted hypo-intensity of the tumor has been shown to be a potential distinctive characteristic for the discrimination of RCC from other renal tumors that are prevalent at this age, especially WTs. Future studies should focus on larger study populations through international collaboration, also exploring innovative techniques such as DWI as a non-invasive biomarker.

## Figures and Tables

**Table 1:** Scan parameters at 1.5-T MRI of the scanned sequences.

Patient nr.	1	2	3	4	5	6
T2-weighted imaging						
Repetition time (ms)	7500	447	1400	454	2457	2457
Echo time (ms)	123	90	92	90	100	100
Slice thickness (mm)	5.5	1.15	4	1.15	5	5
Echo train length	17	85	256	85	39	39
Slicing gap	6.5	1.15	4.4	1.15	5	5
Acquisition matrix	320 × 224	348 × 348	384 × 194	348 × 348	452 × 78	452 × 78
T1-weighted imaging						
Repetition time (ms)	6.3	5.5	4.7	5.4	5.5	5.5
Echo time (ms)	3.1	2.7	2.4	2.7	2.7	2.7
Slice thickness (mm)	5	3	3	3	3	3
Echo train length	1	60	1	60	60	60
Slicing gap	2.5	1.5	*NS*	1.5	1.5	1.5
Acquisition matrix	288 × 192	232 × 233	320 × 170	232 × 233	232 × 233	260 × 261
Diffusion weighted imaging						
Repetition time (ms)	13333	2084	5300	2084	2398	2398
Echo time (ms)	634	72	75	72	73	73
Slice thickness (mm)	6	5	6	5	5	5
Echo train length	1	35	1	35	35	35
Slicing gap	7.2	5	7.2	5	5	5
Acquisition matrix	NS	88 × 70	192 × 153	88 × 70	88 × 70	88 × 70
b values	0/50/600/1000	0/50/200/400/800	0/500	0/50/200/400/800	0/100/1000	0/100/1000

ms = milliseconds; mm = millimeters; NS = not specified.

**Table 2:** Characteristics of the included pediatric patients with RCC.

Patient nr.			1	2	3	4	5	6
Clinical characteristics	Age (months)		184	63	179	109	63	193
	Sex		Female	Female	Female	Male	Female	Male
	Tumor side		Right	Left	Right	Left	Right	Left
	Pre-operative chemotherapy		No	Yes	No	No	Yes	No
	Surgical approach		TN	TN	TN	TN	TN	TN
	Tumor stage		1	1	2	3	1	3
	Biopsy performed		No	No	Yes	No	No	No
Pathology findings	Weight of the specimen (gram)		2100	NS	210	610	753	820
	Tested for MiT-RCC (test)		No	Yes *(FISH)*	Yes *(FISH)*	Yes *(FISH)*	Yes *(FISH, RNA-seq)*	Yes *(RNA-seq)*
	Histopathological subtype		FH-RCC	ccRCC	MiT-RCC	NOS	ccRCC	MiT-RCC
	Genetic analysis		FH-mutation ^d^	NS	NS	NS	None	NS
General tumor characteristics on MRI	Tumor volume (cm^3^)		2191	110	29	353	433	554
	Location of the tumor		Indist	Central	Peripheral	Peripheral	Central	Indist
	Regional lymph nodes		No	No	No	No	No	No
	Shape		Lobulated	Round	Lobulated	Lobulated	Lobulated	Round
	Margins		Well-def	Well-def	Ill-def	Ill-def	Well-def	Well-def
	Pseudocapsule		Yes	Yes	No	No	Yes	Yes
Growth pattern on MRI	Capsule rupture/invasion		No	No	Yes	Yes	No	No
	Infiltrative growth pattern		No	No	Yes	No	No	No
	Venous invasion/Tumor thrombus		No	No	No	No	No	No
MRI characteristics of solid components of the tumor	T2W imaging	Pattern	Hetero	Homo	Homo	Homo	Homo	Hetero
		Intensity	Hypo, Iso	Iso	Hypo	Hypo	Hypo	Hypo
	T1W imaging	Pattern	Hetero	Homo	Homo	Homo	Homo	Hetero
		Intensity	Iso	Iso	Iso	Hypo	Iso	Hypo
	Enhancement, degree and pattern		Strong, homo	Mild, homo	Strong, homo	Strong, homo	NA ^a^	Mild, hetero
	Hemorrhage, degree		No	Yes, ext ^b^	No	Yes, minimal	No	Yes, min ^c^
	Necrosis		No	No	No	No	No	Yes
	Cysts		Yes	Yes ^b^	No	No	No	Yes
	Septation		No	No	No	No	No	No
	Fatty tissue		No	No	No	No	No	No
	Subcapsular fluid		No	No	No	No	No	Yes ^c^
	Increased vascularity		No	No	Yes	Yes	No	Yes
	Median surface ROIs (cm^2^)		4.29	0.45	2.66	9.06	18.14	2.61
	Median ADC value^d^ (×10^−3^ mm^2^/s)		1.20	1.05	0.98	1.20	0.70	0.80

TN = total nephrectomy; NS = not specified; FISH = fluorescence in situ hybridization; RNA-seq = RNA sequencing; RCC = renal cell carcinoma; FH-RCC = fumarate-hydratase-deficient RCC; ccRCC = clear cell type RCC; MiT-RCC = translocation-type RCC; NOS = not otherwise specified; Indist = indistinguishable; def = defined; Hetero = heterogeneous; Homo = homogeneous; ext = extensive; min = minimal. ^a^ No contrast-enhanced diagnostic MRI scan available. ^b^ Hemorrhage of/in the cystic lesion. ^c^ Subcapsular fluid suspected of hemorrhage. ^d^ Hereditary leiomyomatosis and renal cell cancer.

**Table 3:** Review of the literature focusing on MRI characteristics of pediatric and young-adolescent renal cell carcinoma.

Author (Year)	Country	Nr. of Patients	Age (Years)	Sex (M:F)	Histological Subtype	Study Design	Tumor Side (L:R)	Tumor Size (largest Diameter in cm)	Tumor Location	T1-Weighted Imaging Appearance	T2-Weighted Imaging Appearance	Contrast-Enhanced Imaging Appearance	Tumor Composition and Growth Pattern	Necrosis (nr. of Total)	Hemorrhage (nr. of Total)	Vascular Involvement (nr. of Total)	Intra-Tumoral Fat	Regional Lymph Node Involvement/Lymph node Metastases (nr. of Total)	(Distant) Metastases Other Than Lymph Nodes
Norena-Rengifo (2021) [42]	Col	1	12	1:0	RMC	*CR*	1:0	NS	central	inter	hetero, hypo	hypovascular	solid, infiltrative	1	NS	absent	absent	renal hilum, para-aortic	absent
Koetter (2020) [43]	USA	1	16	0:1	P1	*CR*	1:0	17.3	exophytic	NS	NS	hetero	cystic–solid	1	NS	absent	NS	peri-aortic, peri-caval	absent
Schaefer (2017) [44]	USA	1	14	1:0	MiT	*CR*	0:1	5.2	upper pole	homo	hetero	NS	solid	NS	NS	absent	NS	absent	absent
Okabe (2016) [45]	Japan	1	4	1:0	CHR	*CR*	0:1	2.5	NS	hypo	hetero, hyper	NS	well defined	1	NS	NS	NS	NS	NS
Zhou (2016) [46]	China	1	17	1:0	CC ^a^	*CR*	B	0.2–2.0 ^a^	B	hypo	hypo	strong	multiple B ^a^	NS	NS	absent	NS	absent	synchronous CNS hemangioblastoma and pancreatic neuroendocrine tumor
Liu (2014) [24]	China	3	15–33	1:2	MiT	*CR*	1:2	18; 6; 11	cortical	hyper	hetero, hypo	hetero hypo	solid (2); cystic (1); infiltrative (3)	focal (2), central (1)	inter-tumor (3)	absent	NS	regional (2)	absent
Wang (2014) [19]	USA	7 ^b^	13–33	3:4	MiT	*RS*	4:3	3.5–22	medullary (2); medullary cortical (4); exophytic (1)	iso (1); hyper (1); hetero (5)	hypo (1); hyper (1); hetero (5)	hetero: mild (1); moderate (4); marked rim/capsule (2)	irregular (6); not irregular (1); well defined (4); ill defined (3)	7	6	3	NS	regional (4), cervical (1)	absent
Koo (2013) [47]	South Korea	1	28	0:1	MiT	*RS*	0:1	2.7	NS	NS	hetero, hyper	NS	well defined	NS	NS	NS	absent	NS	absent
Dang (2012) [48]	USA	2	18; 31	1:1	MiT	*RS*	0:1 B	8.9; 4.9	NS	hetero, hyper	NS	limited hetero (1); *NS* (1)	NS	1	2	absent	NS	absent	absent
Downey (2012) [49]	USA	2 ^c^	NS	NS	NS	*RS*	NS	NS	NS	hetero, hyper (1); NS (1)	NS	hetero	NS	*NS*	intra-tumoral (1)	NS	NS	NS	NS
Kato (2011) [50]	Japan	1	18	1:0	MiT	*CR*	0:1	4.1	peripheral	NS	hetero, hypo rim, central hyper	delayed peripheral hyper, rim hyper	well demarcated	NS	NS	NS	absent (hemosiderin)	NS	NS
Blitman (2005) [51]	USA	6 (3) ^d^	15–27	3:3	RMC	*RS*	0:6	NS	central	NS	NS	hetero	infiltrative, ill-defined margins	4	intra-tumoral (4); sub-capsular (1)	ipsilateral renal vein (2); encasement vascular pedicle (3)	NS	cervical (6); retroperitoneal (5) ^e^	liver (2); lung (3)
Adachi (2003) [52]	Japan	1	4	1:0	CCP	*CR*	1:0	NS	NS	NS	NS	hyper walls	complicated cyst	NS	cystic (1)	NS	NS	absent	absent

^a^ Multiple (cystic and) bilateral lesions in patient with von Hippel–Lindau disease; ^b^ MRI findings were not specified for each patient separately, so two adult patients (36 and 46 years old) could not be excluded from the overall MRI-data but were not included in the clinical characteristics data; ^c^ Total of nine children but only 2 with MRI scan and no specific details for separate patients (5:4 sex, mean age 12.9 years with a range of 7–17 years, mean maximum diameter 6.2cm (1.5–12.6)); ^d^ Imaging characteristics were not reported separately per patient, leaving no opportunity to extract MRI-specific information. Information displayed is for all 6 patients, based on CT and MRI; ^e^ Retroperitoneal adenopathy was heterogeneous and ranging in volume from small (*n* = 1) or moderate (*n* = 2) to extensive (*n* = 2).; M = male; F = female; L = left; R = right; Col = Colombia; RMC = renal medullary carcinoma; CHR = chromophobe RCC; CC = clear-cell RCC; P1 = papillary type 1 RCC; MiT = translocation-type RCC; CCP = clear-cell papillary type RCC; CR = case report; RS = retrospective cohort study; B = bilateral; homo = homogeneous; hetero = heterogeneous; hypo = hypo-intense; iso = iso-intense; hyper = hyper-intense; inter = intermediate; incr = increase; CNS = central nervous system; NS = not specified.

**Table 4:** Review of the literature focusing on MRI characteristics of translocation-type renal cell carcinoma (MiT-RCC).

Author (Year)	Country	Nr. of Patients	Age (Median Years, Range)	Sex (M:F)	Study Design	Tumor Side (L:R)	Tumor Size (Largest Diameter in cm)	Tumor Location	T1-Weighted Imaging Appearance	T2-Weighted Imaging Appearance	Contrast-Enhanced Imaging Appearance	Diffusion Restriction (ADC value x10^−3^ mm^2^/s)	Tumor Composition and Growth Pattern	Necrosis (nr. of Total)	Hemorrhage (nr. of Total)	Vascular Involvement (nr. of Total)	Intra-Tumoral Fat	Regional Lymph Node involvement/Lymph Node Metastases (nr. of total)	(Distant) Metastases Other than Lymph Nodes
Tohi (2021) [54]	Japan	1	78	1:0	*CR*	R	2.0	posterior	iso	hypo	NS	no restriction ^a^	well circumscribed, no capsule	NS	NS	NS	absent	absent	absent
Dai (2019) [55]	China	16	47.4 (20–76)	9:7	*RS*	9:7	1.7–14.6	endophytic epicenter (14)	hypo (2), iso (5), hyper (9)	hetero (14); hypo (13), iso (6), hyper (2)	hetero (7)	hyper on DWI (b0/500) ((16)	irregular (9), regular (7); complete capsule (11), incomplete capsule (5); solid (11), cystic (2), mixed (3)	NS	5	2	absent	3	retroperitoneal space and liver (1); lung (1)
Gong (2018) [56]	China	2	50; 45	1:1	*CR*	1:1	10.6; 5.2	upper pole (1); lower pole (1)	iso (1), hypo (1)	hypo (2)	hetero (1)	NS	irregular (1)	1	NS	absent	NS	1	absent
Chen (2017) [57]	China	2	46; 30	0:2	*RS*	0:2	7.8; *NS*	NS	hetero iso (2)	hetero (2); hyper (1), hypo (1)	hetero (2)	relatively high signal on DWI (b0/800) (1)	oval (17), irregular (4); solid (4), cystic (1), mixed (16) ^b^	NS	NS	v. renalis (1)	NS	1	liver (1)
Schaefer (2017) [44]	USA	1	14	1:0	*CR*	0:1	5.2	upper pole	homo	hetero	NS	NS	solid	NS	NS	absent	NS	absent	absent
Yu (2016) [58]	China	1	40	1:0	*CR*	0:1	12	NS	iso	hetero hypo-hyper	NS	NS	well defined, irregular	1	patchy (1)	absent	NS	1	absent
D’Antonio (2016) [59]	Italy	1	71	0:1	*CR*	B ^c^	12.0	NS	hetero	hyper	NS	NS	poorly circumscribed (1)	1	1	NS	NS	NS	NS
Liu (2014) [24]	China	4	15–45	1:3	*RS*	1:3	4–18	cortical (4)	hyper (4)	hypo (3), hyper (1)	Hypo	NS	infiltrative (4); solid (3); cystic (1)	focal (3), center (1)	inter-tumor (4)	absent	absent	lymphadenopathy (3)	absent
Wang (2014) [19]	USA	9	13–46	3:6	*RS*	4:5	2–22	medullary (3), medullary cortical (4), exophytic (1), pelvis (1)	iso (1), hyper (3), hetero (5)	hypo (1), hyper (2), hetero (60)	hetero: mild (1), moderate (6), marked rim/capsule (2)	NS	capsule (3); irregular (8); oval (1); well defined (5); ill defined (4)	8	7	4	NS	regional (5), cervical (1)	absent
Koo (2013) [47]	South Korea	2	28; 71	0:2	*RS*	0:2	2.7; 4.6	NS	NS	hetero, hypo (2)	NS	NS	well defined (2)	NS	intra-tumoral (1)	NS	absent	NS	absent
Dang (2012) [48]	USA	2	18; 31		*RS*	0:1 B	8.9; 4.9	NS	hetero, hyper	NS	limited hetero (1); NS (1)	NS	NS	1	2	absent	NS	absent	absent
Razek (2011) [13]	Egypt	4	5–67 ^d^	NS	*PS*	NS	*NS*	NS	NS	NS	NS	mean 1.50 ±0.97 (1.37–1.62) (b0/800)	NS	NS	NS	NS	NS	NS	NS
Kato (2011) [50]	Japan	1	18	1:0	*CR*	0:1	4.1	peripheral	NS	hetero, hypo rim, central hyper	delayed peripheral hyper, rim hyper	NS	well demarcated	NS	NS	NS	absent (hemosiderin)	NS	NS

^a^ Tumor showed no restricted diffusion with a low signal; a fat-poor angiomyolipoma was in the differential diagnosis; ^b^ Total study consisted of 21 patients, of which MRI characteristics were reported for only 2 patients. The tumor composition, shape and growth pattern are, therefore, reported for the total population, mainly based on CT; ^c^ Bilateral tumor, with a right conventional RCC and a left MiT-RCC. Therefore, the characteristics of the MiT-RCC are presented in the table; ^d^ Study with 55 patients, of which 4 had an MiT-RCC. Age was presented for all patients. M = male; F = female; L = left; R = right; CR = case report; RS = retrospective cohort study; PS = prospective cohort study; B = bilateral; homo = homogeneous; hetero = heterogeneous; hypo = hypo-intense; iso = iso-intense; hyper = hyper-intense; inter = intermediate; ADC = apparent diffusion coefficient; DWI = diffusion weighted imaging; incr = increase; NS = not specified.

## Data Availability

Restrictions apply to the availability of these data. The data that support the findings of this study are available in the Supplementary Materials and from the International Society of Pediatric Oncology–Renal Tumor Study Group office following standard access procedures upon reasonable request.

## Supplementary Materials

The following supporting information can be downloaded at https://www.mdpi.com/article/10.3390/cancers15051401/s1, Table S1: Search strategy focusing on MRI characteristics of RCC; Table S2: Observed percentage agreement for dichotomous and categorical characteristics in the case report form for the two observers; Table S3: Median surface of ROI and median ADC values per patient for the two observers.
