# Supplementary material for: MRI Characteristics of Pediatric and Young-Adult Renal Cell Carcinoma: A Single-Center Retrospective Study and Literature Review
Source: Cancers (Basel). 2023 Feb 22;15(5):1401. doi: 10.3390/cancers15051401 (PMC10000563; doi:10.3390/cancers15051401)
Supplement: Supplementary file 1 [file cancers-15-01401-s001.zip › cancers-2185359-supplementary.pdf]

Table S1. Search strategy focusing on MRI-characteristics of RCC.

**PubMed**
"adenocarcinoma of kidney*"[Title/Abstract] OR "adenocarcinoma of the kidney"[Title/Abstract] OR "Grawitz"[Title/Abstract] OR "collecting duct carcinoma*"[Title/Abstract] OR "hypernephroma*"[Title/Abstract] OR "renal cell carcinoma*"[Title/Abstract] OR "carcinoma, renal cell"[MeSH Terms] OR (("carcinoma*"[Title/Abstract] OR "adenocarcinoma*"[Title/Abstract] OR "cancer*"[Title/Abstract]) AND ("Renal"[Title/Abstract] OR "renal cell"[Title/Abstract] OR "nephrotic"[Title/Abstract] OR "nephroid"[Title/Abstract] OR "hypernephro*"[Title/Abstract]))
AND
"magnetic resonance imaging*"[Title/Abstract] OR "MRI"[Title/Abstract] OR "MRIs"[Title/Abstract] OR "fMRI"[Title/Abstract] OR "fMRIs"[Title/Abstract] OR "nmr imaging*"[Title/Abstract] OR "mr tomograph*"[Title/Abstract] OR "mr imaging*"[Title/Abstract] OR "nmr tomograph*"[Title/Abstract] OR "zeugmatograph*"[Title/Abstract] OR "chemical shift imaging*"[Title/Abstract] OR "proton spin tomograph*"[Title/Abstract] OR "magnetization transfer contrast imaging*"[Title/Abstract] OR "spin echo imaging*"[Title/Abstract] OR "Magnetic Resonance Imaging"[MeSH Terms] OR "cross sectional imaging*"[Title/Abstract]
**Embase/Medline**
'adenocarcinoma of kidney*':ti,ab,kw OR 'adenocarcinoma of the kidney':ti,ab,kw OR Grawitz:ti,ab,kw OR 'collecting duct carcinoma*':ti,ab,kw OR hypernephroma*:ti,ab,kw OR 'renal cell carcinoma':ti,ab,kw OR 'renal cell carcinoma'/exp OR ((carcinoma*"ti,ab,kw OR adenocarcinoma*:ti,ab,kw OR cancer*;ti,ab,kw) AND (renal:ti,ab,kw OR 'renal cell':ti,ab,kw OR nephrotic:ti,ab,kw OR nephroid:ti,ab,kw OR hypernephro*:ti,ab,kw))
AND
'magnetic resonance imaging*':ti,ab,kw OR 'MRI':ti,ab,kw OR 'MRIs':ti,ab,kw OR 'fMRI':ti,ab,kw OR 'fMRIs':ti,ab,kw OR 'nmr imaging*':ti,ab,kw OR 'mr tomograph*':ti,ab,kw OR 'mr imaging*':ti,ab,kw OR 'nmr tomograph*':ti,ab,kw OR zeugmatograph*:ti,ab,kw OR 'chemical shift imaging*':ti,ab,kw OR 'proton spin tomograph*':ti,ab,kw OR 'magnetization transfer contrast imaging*':ti,ab,kw OR 'spin echo imaging*':ti,ab,kw OR 'nuclear magnetic resonance imaging'/exp OR 'cross sectional imaging*':ti,ab,kw
**Cochrane**
"adenocarcinoma of kidney*":ti,ab,kw OR "adenocarcinoma of the kidney":ti,ab,kw OR Grawitz:ti,ab,kw OR "collecting duct carcinoma*":ti,ab,kw OR hypernephroma*:ti,ab,kw OR "renal cell carcinoma*":ti,ab,kw OR ((carcinoma*:ti,ab,kw OR adenocarcinoma*:ti,ab,kw OR cancer*:ti,ab,kw) AND (renal:ti,ab,kw OR "renal cell":ti,ab,kw OR nephrotic:ti,ab,kw OR nephroid:ti,ab,kw OR hypernephro*:ti,ab,kw))
AND
"magnetic resonance imaging*":ti,ab,kw OR "MRI":ti,ab,kw OR "MRIs":ti,ab,kw OR "fMRI":ti,ab,kw OR "fMRIs":ti,ab,kw OR "nmr imaging*":ti,ab,kw OR "mr tomograph*":ti,ab,kw OR "mr imaging*":ti,ab,kw OR "nmr tomograph*":ti,ab,kw OR zeugmatograph*:ti,ab,kw OR "chemical shift imaging*":ti,ab,kw OR "proton spin tomograph*":ti,ab,kw OR "magnetization transfer contrast imaging*":ti,ab,kw OR "spin echo imaging*":ti,ab,kw OR "cross sectional imaging*":ti,ab,kw

Table S2. Observed percentage agreement for dichotomous and categorical characteristics in the case report form for the two observers.

Characteristic	Answer options	Observed percent agreement (%)
General tumor characteristics		
Location lesion	*indistinguishable / central / peripheral*	66.7
Regional LNs	*yes / no*	100
Shape	*lobulated/round / irregular*	83.3
Margins	*well-defined / ill-defined*	66.7
Pseudo(capsule)	*yes / no*	66.7
Growth pattern		
Capsule rupture/Capsule invasion	*yes / no*	50
Infiltrative growth pattern	*yes / no*	83.3
Venous invasion/Tumor thrombus	*yes / no*	100
MRI characteristics of solid components of the tumor		
T2W pattern	*heterogeneous / homogeneous / other*	100
T2W intensity	*hyperintense / hypointense / isointense*	66.7
T1W pattern	*heterogeneous / homogeneous / other*	100
T2W pattern	*hyperintense / hypointense / isointense*	33.3
Degree of enhancement	*poor / mild / strong*	66.7
Enhancement pattern	*homogeneous/peripheral to central progressive/band-like areas of late or non-enhancement / heterogeneous / other*	100
Hemorrhage	*yes / no*	83.3
Necrosis	*yes / no*	100
Cysts	*yes / no*	83.3
Septation	*yes / no*	66.7
Fatty tissue	*yes / no*	100
Subcapsular fluid	*yes / no*	83.3
Increased vascularity	*yes / no*	66.7

T2W = T2-weighted; T1W = T1-weighted.

Table S3. Median surface of ROI and median ADC-values per patient for the two observers.

Patient nr.	Observer	Diffusion restriction	Number of ROIs	Median surface (cm^2, range)	Median ADC-value ($*10^{-3}$ mm^2/s, range)
1	1	Yes	2	4.29 (3.99-4.59)	1.20 (1.10-1.30)
	2	Yes	4	2.11 (1.2-2.98)	1.33 (1.13-1.37)
2	1	Yes	2	0.45 (0.32-0.57)	1.05 (0.90-1.20)
	2	Yes	2	0.28 (0.22-0.33)	0.94 (0.76-1.11)
3	1	Yes	2	2.66 (2.51-2.81)	0.98 (0.96-1.00)
	2	Yes	3	1.32 (1.03-1.58)	1.00 (0.99-1.01)
4	1	Yes	3	9.06 (6.55-24.83)	1.20 (1.20-1.20)
	2	Yes	4	12.70 (12.00-14.10)	1.14 (1.12-1.18)
5	1	Yes	2	18.14 (9.68-26.59)	0.70 (0.70-0.70)
	2	Yes	4	19.40 (2.7-21.6)	0.69 (0.67-0.70)
6	1	Yes	4	2.61 (2.35-3.67)	0.80 (0.80-0.90)
	2	Yes	4	1.68 (0.96-2.70)	0.80 (0.76-0.84)

ROI = region of interest; ADC = apparent diffusion coefficient.
